# Positive effects of rutin on egg quality, lipid peroxidation and metabolism in post-peak laying hens

**DOI:** 10.3389/fvets.2024.1426377

**Published:** 2024-05-30

**Authors:** Leizheng Zhang, Jiangang Gong, Lin Xi, Bowen Yang, Yanshuang Hao, Haihua Zhang, Zhihua Feng, Qian Li

**Affiliations:** ^1^College of Animal Science and Technology, Hebei Agricultural University, Baoding, China; ^2^College of Food Science and Technology, Hebei Agricultural University, Baoding, China; ^3^Department of Animal Science, North Carolina State University, Raleigh, NC, United States; ^4^College of Animal Science and Technology, Hebei Normal University of Science and Technology, Qinhuangdao, China; ^5^Hebei Institute of Animal Husbandry and Veterinary Medicine, Baoding, China

**Keywords:** rutin, post-peak laying hen, egg quality, peroxidation, metabolism

## Abstract

Excessive fat deposition due to impaired fat metabolism in chickens is a major problem in the poultry industry. Nutritional interventions are effective solutions, but current options are limited. A safe phytochemical, rutin, has shown positive effects in animals, but its effect on lipid metabolism in poultry remains unknown. Hence, this study is to investigate the effects of rutin on egg quality, serum biochemistry, fat deposition, lipid peroxidation and hepatic lipid metabolism in post-peak laying hens. A total of 360 Taihang laying hens (49-week-old) were randomly divided into five groups and fed a basal diet (control group, 0%) and a basal diet supplemented with 300 (0.03%), 600 (0.06%), 900 (0.09%), and 1,200 (0.12%) mg rutin/kg feed, respectively. The results showed that eggshell strength was significantly (*p* < 0.05) higher in the dietary rutin groups, whereas yolk percentage (*p* < 0.05), total cholesterol (TC) (*p* < 0.01) and yolk fat ratio (*p* < 0.01) decreased linearly (*p* < 0.05) in the dietary rutin groups. Importantly, dietary rutin reduced serum triglyceride (TG) and TC levels, decreased abdominal lipid deposition and liver index (*p* < 0.05), and which concomitantly decreased hepatic lipid (TG, TC, and free fatty acid) accumulation (*p* < 0.05). An increase (*p* < 0.05) in total antioxidant capacity and superoxide dismutase activity and a decrease (*p* < 0.05) in malondialdehyde levels were also found. At the same time, the activities of hepatic lipase, acetyl-CoA carboxylase and malic enzyme in the liver were decreased (*p* < 0.05). Dietary rutin also increased (*p* < 0.05) the expression of fatty acid oxidation-related genes (carnitine palmitoyl transferase 1, peroxisome proliferator-activated receptor α, farnesoid X receptor). Additionally, it decreased fatty acid synthesis genes (sterol regulatory element binding protein-1c, acetyl-CoA carboxylase α, stearoyl-CoA desaturase 1) (*p* < 0.05). In conclusion, the addition of rutin (0.06–0.12%) to the diet improved the fat metabolism and increased liver antioxidant capacity in post-peak laying hens, and these positive changes improved egg quality to some extent.

## 1 Introduction

One of the most significant challenges currently facing the laying hen industry is the decline in egg quality observed during the latter stages of the peak laying period (1). This phenomenon can be attributed to various factors, such as aging-related changes in the reproductive system (2), decreases in metabolic capacity (3), and changes in the dominant intestinal microbiota (4), etc. Among them, lipid metabolism is an indispensable part of egg formation, and is also closely related to its molecular role and physiological characteristics in liver, the primary lipid metabolic organ of laying hens (5). Liver stress during aging can worsen lipid metabolism disorders, and raise the likelihood of related metabolic diseases (6). Furthermore, in large-scale caged environments where laying hens have a limited mobility and reduced exercise, the excess unutilized energy is converted to fat and deposited in the abdomen and major metabolic organs (7). Consequently, how to mitigate some of the adverse effects of fat metabolism disorders on laying hens has become a pressing issue in large-scale egg production.

In accordance with the tenets of healthy consumption and the policies of numerous countries, antibiotics and chemical synthetic drugs are prohibited (or subject to restrictions) for use in the treatment of metabolic diseases in poultry (8). Instead, natural herbs and their extracts with biological and medical activities have attracted great attention and are being studied extensively for the potential applications in poultry industry. This is due to their natural, safe and broad-spectrum characteristics (9).

Rutin, also known as vitamin P or 3,3′,4′,5,7-pentahydroxy-flavon3-(o-rhamnosylglucoside), is a flavonoid glycoside produced by plants as part of their defense system (10). A versatile exogenous supplement, rutin is found in high concentrations in rue leaves, orange peels, tomatoes, and buckwheat flowers (11). The antioxidant (12), antimicrobial (13), anticancer (14), antidiabetic (15), cardiovascular protection (16) as well as analgesic (17) effects of the compound have been well established *in vitro* tests and animal experiments. In addition of the antioxidant, prebiotics and medical activity mentioned above, rutin also counteracts mitochondrial damage, and acts as a modulator of lipid metabolic disorders (18).

However, the biological role of rutin in lipid metabolism has not been studied extensively in farm animals such as poultry. Results from a study with broilers showed that supplementation of rutin (1 g /kg) in feed enhanced the antioxidant capacity, and reduced lipogenesis, thereby decreasing fat deposition and lipid levels in blood (19). In light of previous studies, our hypothesis was that administration of rutin to aged laying hens could improve on egg quality through alterations in antioxidant status and lipid metabolism. To test our hypothesis, we investigated the effects of dietary supplementation with different doses of rutin on lipid deposition, peroxidation and metabolism in post-peak laying hens. The potential mechanisms of rutin action egg quality via modifying lipid metabolism were discussed.

## 2 Materials and methods

### 2.1 Birds, experimental design, and management

Taihang chicken, a local breed in China, was selected as an experimental bird. Compared with other commercial breeds, it is more resistant to stress and has a lower egg production rate and body weight. Briefly, 360 healthy 49-week-old Taihang laying hens (1.40 ± 0.25 kg, no significant difference in egg production rate) were provided by Jinkai Animal Husbandry Co., LTD (Hebei, China). The hens were randomly divided into five treatment groups. Each group contained six replicates, and 12 hens per replicate. The first group was the control group (0%), which was fed only the basal diet, while the other four groups were supplemented with 0.3 (0.03%), 0.6 (0.06%), 0.9 (0.09%), and 1.2 (0.12%) g/kg rutin in the basal diet, respectively. The experiment lasted 56 d. All the layers were housed in environmentally controlled cages for 7 days to habituate to the experimental environment before the start of the experiment. These birds were hatched on the same hatchery, and at various stages prior to their use in this experiment, were housed in the same environment and received the same diet, vaccination, and decreasing light program.

The experimental diet (Table 1) was formulated based on the Chicken Feeding Standards of China (NY/T33-2004) and the NRC (20) recommendations. The nutritional levels of crude protein (GB/T 6432-2018), calcium (GB/T 6436-2018), and total phosphorus (GB/T 6437-2018) in the diets were analyzed using methods provided by the Chinese Standardization Administration. Rutin is light green powder, purchased from Yuanbeichun Bioengineering Co., Ltd. (Shanxi, China), extracted by hot-water immersion, with a purity greater than 95% (determined by HPLC). The dose of rutin in the diets was selected based on our unpublished pre-experimental data combined with the data reported in previous studies (19, 21).

**Table 1:** Composition and nutrient levels of the basal diet (% air-dry basis).

Items	Content (%)
**Ingredients**	
Corn	64.81
Soybean meal	19.83
Wheat bran	4.95
Soybean oil	1.00
CaHPO4	1.16
NaCl	0.25
Limestone	7.00
Premix^a^	1.00
Total	100.00
**Nutrient levels** ^b^	
ME (Kcal/kg)	2750.89
CP (%)	15.98
Ca (%)	3.20
Total P (%)	0.58
Available P (%)	0.38
Lysine (%)	0.80
Methionine (%)	0.34

a The premix provided the following per kg of the diet: Zn (as zinc sulfate), 80.00 mg; Fe (as ferrous sulfate), 60.00 mg; Mn (as manganese sulfate), 60.00 mg; Cu (as copper sulfate), 8.00 mg; I (as potassium iodide), 0.35 mg; Se (as sodium selenite), 0.30 mg; vitamin A, 2000.00 IU; vitamin D_3_, 1600.00 IU; vitamin E_3_, 5.00 IU; vitamin K, 0.50 IU; vitamin B_1_, 0.80 IU; vitamin B_2_, 2.50 mg; vitamin B_6_, 3.00 mg; nicotinic acid, 20.00 mg; D-pantothenic acid, 2.20 mg; folic, 0.25 mg; biotin, 0.10 mg; methionine, 0.80 g; lysine, 1.90 g.

b Nutrient level: CP, Ca, total P were analyzed values. Others were calculated values.

During the experimental period, all layers were housed in three-tier upright rearing cages with one bird per cage (0.16 m^2^/bird). The temperature (25 ± 2°C), relative humidity (60 ± 5%) and light–dark cycle (15.5 L: 8.5 D) in the hen house were kept within safe and controllable limits. Hens were allowed to eat and drink freely.

### 2.2 Egg quality

Three eggs were randomly selected from each replicate (same for yolk lipid accumulation) to determine egg quality on day 56 of the experiment. The egg’s long and short diameters were measured using a coefficient measuring instrument (NFN385, FHK Corp., Tokyo, Japan), and the egg shape index was the ratio of the long and short diameters. Eggshell strength was measured using an egg force reader (EFR-01, ORKA Technology Co., Ltd., Herzliya, Israel). Eggshell thickness tester (ESTG-01, ORKA Technology Co., Ltd., Herzliya, Israel) was used to determine the eggshell thickness. Egg multitester (model EA-01, ORKA Technology Co., Ltd., Herzliya, Israel) was used to determine the Haugh unit and yolk color.

### 2.3 Sample collection

On the morning of day 56, after weighing, one laying hen was randomly selected from each replicate and euthanized by cervical dislocation after blood samples (about 8 mL, collected in two batches) was collected from the wing vein. Blood samples were centrifuged at 3,000 × g for 15 min at 4°C, and serum was collected. The experimental birds were then dissected (total 30 hens), and the liver, spleen, and intestines were removed and weighed. The liver samples were collected into three 2 mL cryotubes and quickly frozen in liquid N_2_. The frozen samples were stored at −80°C freezer for further analysis.

### 2.4 Serum biochemical indices

After the serum samples were rewarmed at room temperature, the levels of total cholesterol (TC), triglyceride (TG), high-density lipoprotein cholesterol (HDL-C), low-density lipoprotein cholesterol (LDL-C), as well as the activities of aspartate transaminase (AST) and alanine transaminase (ALT) were determined using the kits purchased from the Nanjing Jiancheng Bioengineering Institute (Nanjing, China).

### 2.5 Organ index and the percentage of abdominal fat

The ratio of the weight of an organ or abdominal fat to the live body weight, expressed as a percentage, is used to determine the organ index and the percentage of abdominal fat (PAF).

### 2.6 Egg yolk and hepatic lipid accumulation

The fresh liver samples and homogenized egg yolk were freeze-dried in a vacuum freeze-dryer for 72 h and then ground into powder. The fat content of the egg yolk and liver was determined using the Soxhlet extractor method on 500 mg of lyophilized powder.

Fresh liver samples (about 200 mg) were homogenized with pre-cooled saline (1 g liver: 9 mL saline) on ice, while lyophilized egg yolk powder (about 100 mg) was homogenized with pre-cooled anhydrous ethanol (1 g egg yolk powder: 9 mL ethanol) due to the high-fat content. The homogenates were centrifuged at 4,000 × g for 15 min at 4°C, to obtain the supernatant for measurement. The TC, TG and free fatty acid (FFA) levels in liver and egg yolk were determined in supernatant, using kits purchased from Nanjing Jiancheng Bioengineering Institute (Nanjing, China). The protein content in the liver samples was determined using the BCA protein assay kit (Nanjing Jiancheng Bioengineering Institute, Nanjing, China) to normalize TG, TC, and FFA content in the liver.

### 2.7 Hepatic lipid peroxidation

Fresh liver sample was homogenized in cold saline with a ratio of 1:9 (w/v) and the homogenate were centrifuged at 3,500 × g for 15 min at 4°C. The supernatants were obtained after the centrifugation, and used for the biological assays associated with lipid peroxidation. Hepatic total antioxidant capacity (T-AOC), superoxide dismutase (SOD), glutathione peroxidase (GSH-Px), and malondialdehyde (MDA) were determined using kits purchased from Nanjing Jiancheng Bioengineering Institute (Nanjing, China). Similar to the previous section, the results of lipid peroxidation were normalized to the protein concentration in each sample.

### 2.8 Hepatic fat metabolizing enzyme activity

As described above, hepatic fat metabolizing enzyme activity assay also requires preparation of liver tissue supernatant. Liver lipoprteinlipase (LPL) and hepaticlipase (HL) activities were determined using kits from Nanjing Jiancheng Bioengineering Institute (Nanjing, China), and liver acetyl CoA carboxylase (ACC), fatty acid synthase (FAS) and malic enzyme (ME) activities were determined using ELISA kits from Jiangsu Meimian Industrial Co., Ltd. (Jiangsu, China). Of these, the outcomes of LPL and HL must be standardized in relation to the total protein concentration in the supernatant.

### 2.9 Gene expression

Total RNA was extracted from the liver samples according to the instructions of RNAiso Plus (Takara, Beijing, China) and then reverse-transcribed into cDNA using the Prime Script RT kit (Takara, Beijing, China). All primers (Table 2) are designed by Oligo 7, and synthesized by Genewiz Technology (Suzhoui, China) Co., Ltd. after verification of primer specificity. Real-Time PCR amplification of cDNA was performed using the SYBR Premix Ex Taq II kit (Takara, Beijing, China). The PCR reaction volume was 20 μL, including 10 μL of SYBR premix Ex Taq (2×), 2 μL of cDNA template, 0.4 μL of forward and reverse primers, 0.4 μL of ROX reference dye (50×), 6.8 μL of RNase-free water. PCR amplification was performed at 95°C for 30 s, followed by 95°C for 5 s and 60°C for 30 s, for a total of 40 cycles. The relative mRNA expression of each target gene was calculated by the 2^−ΔΔCt^ method using β-actin as an internal reference.

**Table 2:** Primer sequences of genes.

Genes^a^	Primer sequence (5′-3′)	Product size/bp	GenBank accession number
β-actin	F: GTCCACCGCAAATGCTTCTA	104	NM_205518.2
	R: AGCCATGCCAATCTCCGTCTT		
*FXR*	F: GAGTCAGTGGAAAGGCTTCAGGAG	146	NM_001396910.1
	R: ATCTCTGCGTGGTGGTGGTTAAAC		
*PPARα*	F: CTTGTGAAGGTTGTAAGGGTT	146	NM_001001464.1
	R: CATTCCAACTGAAAGGCAC		
*FAS*	F: TCCTTGGTCTTCGTGACG	163	NM_205155.4
	R: CGCAGTTTCTTGATGGTGAG		
*ACCα*	F: AATGGCAGCTTTGGAGGTGT	137	XM_046929958.1
	R: TTCTGTTTGGGTGGGAGGTG		
*CPT-1*	F: AACCCTTGACACAACTGGCT	96	XM_046918284.1
	R: GTGACGATAAGGGCAACCCA		
*SREBP-1c*	F: GTCGGCGATCCTGAGGAA	105	AY029224
	R: CTCTTCTGCACGGCCATCTT		
*SCD1*	F: CACATGGCTTGGCTGCTGGTAC	117	NM_204890.2
	R: CTTGTAGTATCTCCGCTGGAACATCAC		

a FXR, farnesoid X receptor; PPARα, peroxisome proliferator-activated receptor α; FAS, fatty acid synthase; ACCα, acetyl CoA carboxylase α; CPT-1, carnitine palmityl transferase I; SREBP-1c, sterol regulatory element binding protein-1c; SCD1, stearoyl-CoA desaturase 1.

### 2.10 Statistical analyses

SPSS 26.0 statistical software (version 26.0 for Windows, SPSS Inc., Chicago) was used for data analysis. ANOVA in GLM procedure was used to analyze variance, and linear and quadratic *p*-values were also calculated using orthogonal contrasts. A probability of *p* < 0.05 was considered to be statistically significant and the tendency of significant difference was assessed when 0.05 < *p* < 0.10. Duncan’s test was used for *post-hoc* multiple comparisons. Results were shown as means. The overall standard error of mean (SEM) was given.

## 3 Results

### 3.1 Egg quality

As shown in Table 3, there were no (*p* > 0.05) effects of rutin supplementation on egg shape index, eggshell thickness, Haugh unit, yolk color and egg yolk TC. However, a quadratic response of the eggshell strength to rutin concentration was detected (*p* < 0.01). The highest strength was observed in the group supplemented with 0.06% of dietary rutin, and the strength was higher than the control group (*p* < 0.05). In addition, a linear response to dietary rutin supplemented levels was detected for egg shape index (*p* < 0.05), yolk percentage (*p* < 0.05), egg yolk TG (*p* < 0.01) and egg yolk fat ratio (*p* < 0.01). The egg shape index increased with the increase of rutin supplementation in the diet, but the yolk percentage, yolk TG and yolk fat ratio decreased with the increase of rutin in the diet. The addition of rutin to the diet resulted in a mean reduction of 10% in the egg yolk percentage compared to the control group (*p* < 0.01). While the TG levels and fat ratio of egg yolks decreased on average by 27% (*p* < 0.05) and 9% (*p* < 0.005) from the groups with dietary supplemented rutin higher than 0.09%, as compared with the control group.

**Table 3:** Effect of rutin on the egg quality in post-peak laying hens.

Items^1^	Rutin levels					SEM	Statistics		
	0%	0.03%	0.06%	0.09%	0.12%		*P*_anova_	*P*_linear_	*P*_quadratic_
Egg shape index	1.32	1.32	1.33	1.35	1.34	0.01	0.187	0.030	0.817
Eggshell strength (N)	39.66^b^	44.51^ab^	47.54^a^	43.63^ab^	42.93^ab^	0.81	0.041	0.312	0.007
Eggshell thickness (mm)	0.32	0.31	0.33	0.32	0.31	0.00	0.360	0.801	0.148
Haugh unit	53.37	52.51	53.68	53.77	53.68	0.89	0.992	0.771	0.953
Yolk percentage (%)	0.42^a^	0.37^b^	0.39^b^	0.37^b^	0.38^b^	0.01	0.006	0.016	0.023
Egg yolk color	10.56	11.28	11.28	11.67	11.33	0.16	0.239	0.076	0.183
Egg yolk TG (μmol/g)	119.85^a^	121.98^a^	104.36^ab^	90.39^b^	84.00^b^	4.35	0.013	<0.001	0.683
Egg yolk TC (μmol/g)	32.47	29.51	29.72	27.78	26.46	3.71	0.365	0.066	0.891
Egg yolk fat ratio (%)	46.43^a^	45.63^a^	45.03^a^	42.35^b^	42.25^b^	0.49	0.005	<0.001	0.838

1 TG, triglyceride; TC, total cholesterol.

^a, b^In the same row, different lowercase letters represent significant differences (*p* < 0.05). Values are the mean and standard error of the mean, with the mean being the result of 6 replications of each treatment (*n* = 6).

### 3.2 Serum biochemical indices

Dietary supplementation of the rutin had impacts on serum TG, TC, and HDL-C (Table 4). Dietary rutin decreased serum TG as compared to control, but the decrease was greater from the diet with 0.12% rutin than diets with 0.03, 0.06, and 0.09% (*p* < 0.01). A linear response of serum TG content to the dose of rutin supplemented in the diet was detected (*p* < 0.05). The serum TC and HDL-C were changed in the hen fed diets supplemented with 0.03 and 0.06% of rutin, but the TC reduction was greater in hens with dietary 0.06% than 0.03% of rutin (*p* < 0.05). No differences were detected between hens fed diets with 0.09 and 0.12% of rutin and control hens (*p* > 0.05). A significant quadratic response of serum TC and HDL-C to dietary rutin levels was detected (*p* < 0.05). There was no significant effect of dietary rutin on either serum LDL-C levels, AST and ALT activities (*p* > 0.05), but a quadratic tendency in response of AST to dietary rutin levels was observed (*p* = 0.057).

**Table 4:** Effect of rutin on serum biochemical indices in post-peak laying hens.

Items^1^	Rutin levels					SEM	Statistics		
	0%	0.03%	0.06%	0.09%	0.12%		*P*_anova_	*P*_linear_	*P*_quadratic_
TG (mmol/L)	23.21^a^	18.95^b^	19.21^b^	19.36^b^	14.40^c^	0.63	<0.001	<0.001	0.638
TC (mmol/L)	4.86^a^	3.47^bc^	2.83^c^	3.96^ab^	4.44^ab^	0.20	0.005	0.786	<0.001
HDL-C (mmol/L)	2.51^b^	2.78^a^	2.88^a^	2.65^ab^	2.74^ab^	0.04	0.024	0.173	0.020
LDL-C (mmol/L)	1.35	1.38	1.27	1.24	1.24	0.05	0.869	0.345	0.972
AST (U/L)	23.61	21.34	21.23	22.76	23.02	0.41	0.267	0.932	0.057
ALT (U/L)	6.09	5.65	5.59	5.79	5.63	0.19	0.939	0.608	0.636

1 TG, triglyceride; TC, total cholesterol; HDL-C, high-density lipoprotein cholesterol; LDL-C, low-density lipoprotein cholesterol; AST, aspartate transaminase; ALT, alanine transaminase.

^a, b, c^In the same row, different lowercase letters represent significant differences (*p* < 0.05). Values are the mean and standard error of the mean, with the mean being the result of 6 replications of each treatment (*n* = 6).

### 3.3 Organ index and the percentage of abdominal fat

As shown in Table 5, supplementation of rutin to the diet had no effect on the final body weight (FBW), spleen and intestine index (percentage of body weight) in the laying hens and no differences were detected between the treatment groups (*p* > 0.05). However, supplementing rutin in the diet reduced PAF and liver index (*p* < 0.05). The PAF from the fed with 0.06 and 0.12% of rutin and liver index from hens fed with 0.03 and 0.09% of rutin were on average 28 and 9% lower than that from the control hens (*p* < 0.05). In general, the PAF (*p* < 0.005) and liver index (*p* < 0.05) reduced linearly with the increase of dietary rutin addition. A significant quadratic response of liver index to dietary rutin level was detected also (*p* < 0.05).

**Table 5:** Effect of rutin on organ index and the percentage of abdominal fat in post-peak laying hens.

Items^1^	Rutin levels					SEM	Statistics		
	0%	0.03%	0.06%	0.09%	0.12%		*P*_anova_	*P*_linear_	*P*_quadratic_
FBW (kg)	1.52	1.53	1.55	1.43	1.59	0.037	0.755	0.881	0.608
PAF (%)	4.64^a^	4.07^ab^	3.51^b^	3.90^ab^	3.14^b^	0.156	0.018	0.002	0.614
Liver (%)	1.81^a^	1.69^b^	1.70^ab^	1.61^b^	1.71^ab^	0.020	0.027	0.029	0.033
Spleen (%)	0.12	0.11	0.12	0.11	0.11	0.005	0.923	0.453	0.792
Intestine (%)	4.04	4.19	4.16	4.37	4.04	0.134	0.940	0.853	0.539

1 FBW, final body weight; PAF, the percentage of abdominal fat.

^a, b^In the same row, different lowercase letters represent significant differences (*p* < 0.05). Values are the mean and standard error of the mean, with the mean being the result of 6 replications of each treatment (*n* = 6).

### 3.4 Hepatic lipids accumulation

Dietary supplementation of rutin had a great impact on the hepatic lipid contents in hens (Table 6). The liver fat ratio (fat percentage in liver) in hens was on average 26% lower from 0.09 and 0.12% of rutin groups than the control group (*p* < 0.001). The total TG from 0.06 and 0.12% of rutin groups, TC from 0.06% of rutin group and FFA from 0.09 and 0.12% of rutin group were reduced on average by 17% (*p* < 0.05), 16% (*p* < 0.05) and 11% (*p* < 0.01) respectively compared to the control group. Significant linear responses of hepatic lipid ratio (*p* < 0.01), TG (*p* < 0.05), and FFA (*p* < 0.001) to dietary rutin levels were detected. A quadratic response of hepatic TC to dietary rutin levels was observed (*p* < 0.005).

**Table 6:** Effect of rutin on liver fat accumulation in post-peak laying hens.

Items^1^	Rutin levels					SEM	Statistics		
	0%	0.03%	0.06%	0.09%	0.12%		*P*_anova_	*P*_linear_	*P*_quadratic_
Fat ratio (%)	14.56^a^	12.63^ab^	13.10^a^	10.95^b^	10.65^b^	0.38	<0.001	<0.001	0.803
TG (mmol/g prot)	0.49^a^	0.48^ab^	0.40^c^	0.45^abc^	0.41^bc^	0.01	0.033	0.020	0.467
TC (mmol/g prot)	0.19^a^	0.20^a^	0.16^b^	0.18^a^	0.18^a^	0.01	0.002	0.679	0.002
FFA (μmol/g prot)	184.56^a^	178.23^ab^	176.35^ab^	167.43^bc^	161.64^c^	2.35	0.008	<0.001	0.716

1 TG, triglyceride; TC, total cholesterol; FFA, free fatty acid.

^a, b, c^In the same row, different lowercase letters represent significant differences (*p* < 0.05). Values are the mean and standard error of the mean, with the mean being the result of 6 replications of each treatment (*n* = 6).

### 3.5 Hepatic lipid peroxidation

Dietary supplementation of rutin significantly impacted on hepatic T-AOC (*p* < 0.005) and SOD (*p* < 0.01) activities, and MDA (*p* < 0.005) level in liver of hens (Table 7). The activities of T-AOC and SOD linearly increased with the increase in dietary rutin levels (*p* < 0.001), while the MDA decreased linearly with the increase of dietary rutin levels (*p* < 0.001). The hepatic T-AOC and SOD activities were on average 21 and 10% higher from hens fed diets with 0.06, 0.09 and 0.12% of rutin than from the control group of hens. While the hepatic MDA level was on average 44% (*p* < 0.01) lower from hens fed diets with rutin than the hens in control group. In addition, a significant quadratic response of T-AOC activity and MDA level to the dietary rutin levels was detected (*p* < 0.05). Supplementation of rutin had no effect on hepatic GSH-Px activity (*p* > 0.1).

**Table 7:** Effect of rutin on liver lipid peroxidation in post-peak laying hens.

Items^1^	Rutin levels					SEM	Statistics		
	0%	0.03%	0.06%	0.09%	0.12%		*P*_anova_	*P*_linear_	*P*_quadratic_
T-AOC (U/mg prot)	5.71^c^	6.29^bc^	6.72^ab^	7.29^a^	6.74^ab^	0.15	0.004	0.001	0.040
SOD (U/mg prot)	217.78^c^	227.87^bc^	235.42^ab^	245.01^a^	240.33^ab^	2.81	0.010	<0.001	0.174
GSH-Px (U/mg prot)	75.48	78.71	76.23	77.55	79.23	1.30	0.895	0.521	0.952
MDA (nmol/mg prot)	1.26^a^	1.04^b^	0.85^b^	0.89^b^	0.87^b^	0.04	0.002	0.001	0.025

1 T-AOC, total antioxidant capacity; SOD, superoxide dismutase; GSH-Px, glutathione peroxidase; MDA, malondialdehyde.

^a, b, c^In the same row, different lowercase letters represent significant differences (*p* < 0.05). Values are the mean and standard error of the mean, with the mean being the result of 6 replications of each treatment (*n* = 6).

### 3.6 Hepatic fat metabolizing enzyme activity

As shown in Table 8, supplementation of dietary rutin decreased the enzyme activity of HL, ACC, and ME (*p* < 0.05) and tended to decrease the activity of FAS (*p* = 0.06) in liver. The decrease in activity of HL (*p* < 0.005) and ME (*p* < 0.05) was linearly with the increase of rutin levels in the diet, while the decrease in activity of ACC was quadratically with the increase in dietary rutin levels (*p* < 0.05). Supplementation of dietary rutin had no effect on the LPL activity (*p* > 0.05), but a linear response was observed (*p* = 0.018). The HL and ME activity were lower on average 11 and 10% from hens fed diet with 0.06, 0.09, and 0.12% of rutin than the hens in control group, respectively. The lowest activity of ACC was observed in hens fed with 0.06% of dietary rutin (*p* < 0.05).

**Table 8:** Effect of rutin on liver fat metabolizing enzyme activities in post-peak laying hens.

Items^1^	Rutin levels					SEM	Statistics		
	0%	0.03%	0.06%	0.09%	0.12%		*P*_anova_	*P*_linear_	*P*_quadratic_
LPL (U/mg prot)	0.29	0.27	0.27	0.26	0.24	0.01	0.196	0.018	0.936
HL (U/mg prot)	0.48^a^	0.45^ab^	0.43^b^	0.44^b^	0.41^b^	0.01	0.016	0.002	0.294
ACC (U/L)	146.19^a^	144.70^a^	139.21^b^	143.55^a^	144.06^a^	0.72	0.019	0.228	0.013
FAS (U/mL)	2254.98	2208.51	2112.62	2119.00	2113.65	20.12	0.060	0.008	0.239
ME (mIU/L)	2745.37^a^	2654.33^ab^	2496.31^bc^	2506.54^bc^	2387.96^c^	37.34	0.011	<0.001	0.669

1 LPL, lipoprteinlipase; HL, hepatic lipase; ACC, acetyl-CoA carboxylase; FAS, fatty acid synthase; ME, malic enzyme.

^a, b, c^In the same row, different lowercase letters represent significant differences (*p* < 0.05). Values are the mean and standard error of the mean, with the mean being the result of 6 replications of each treatment (*n* = 6).

### 3.7 Expression of lipid metabolism-related genes

As shown in Table 9, the mRNA abundance of carnitine palmitoyl transferase I (*CPT-1*) and peroxisome proliferator-activated receptor α (*PPARα*) and famesoid X receptor (*FXR*) increased in the liver of hens fed diets with supplementation of rutin. The increase in expressions of *CPT-1* (*p* < 0.001) and *PPARα* (*p* < 0.05) was linearly with the increase in dietary supplementation of rutin. A significant quadratic response of the gene expression of *CPT-1* (*p* < 0.001) and *PPARα* (*p* < 0.005) to the dietary rutin was detected also. The abundance of *CPT-1* and *PPARα* was greater from hens with 0.06% dietary rutin than with 0.03, 0.09, and 0.12% dietary rutin. In addition, supplementation of dietary rutin increased *FXR* abundance, but the increase was greater from hens fed diet with 0.12% rutin than hens fed the control diet (*p* < 0.05). A significant quadratic relationship between the gene expression and dietary rutin levels was detected (*p* < 0.001).

**Table 9:** Effect of rutin on the expression of lipid metabolism-related genes in post-peak laying hens.

Items^1^	Rutin levels					SEM	Statistics		
	0%	0.03%	0.06%	0.09%	0.12%		*P*_anova_	*P*_linear_	*P*_quadratic_
**Fatty acid oxidation**									
*CPT-1*	1.00^c^	1.27^b^	1.53^a^	1.43^ab^	1.32^b^	0.04	<0.001	<0.001	<0.001
*PPARα*	1.00^c^	1.45^ab^	1.73^a^	1.61^ab^	1.34^b^	0.06	<0.001	0.013	0.004
*FXR*	1.00^b^	1.31^a^	1.29^a^	1.26^a^	1.10^ab^	0.04	0.018	0.522	<0.001
**Fatty acid synthesis**									
*SREBP-1c*	1.00^a^	0.89^ab^	0.86^ab^	0.73^b^	0.77^b^	0.06	0.022	0.002	0.354
*ACCα*	1.00^a^	0.91^a^	0.66^b^	0.46^c^	0.66^b^	0.04	<0.001	0.002	0.004
*SCD1*	1.00^ab^	1.13^a^	0.66^c^	0.57^c^	0.83^bc^	0.05	<0.001	0.003	0.059
*FAS*	1.00	0.95	0.89	0.95	1.07	0.04	0.760	0.687	0.220

1 CPT-1, carnitine palmityl transferase I; PPARα, peroxisome proliferator-activated receptor α; FXR, farnesoid X receptor; SREBP-1c, sterol regulatory element binding protein-1c; ACCα, acetyl-CoA carboxylase α; SCD1, stearoyl-CoA desaturase 1; FAS, fatty acid synthase.

^a, b, c^In the same row, different lowercase letters represent significant differences (*p* < 0.05). Values are the mean and standard error of the mean, with the mean being the result of 6 replications of each treatment (*n* = 6).

Contrary to increasing expression of genes associated with fatty acid oxidation, addition of rutin in the diet reduced the expression of genes associated with fatty acid synthesis. The abundance of sterol regulatory element binding protein-1c (*SREBP-1*c) was on average 25% lower from hens with dietary 0.09 and 0.12% rutin than with the control diet (*p* < 0.05). The abundance of acetyl-CoA carboxylase α (*ACCα*) was on average 41% lower from hens with dietary 0.06, 0.09 and 0.12% rutin than with the control diet (*p* < 0.001) and the abundance of stearoyl-CoA desaturase 1 (*SCD1*) was on average 39% lower from hens with 0.06 and 0.09% dietary rutin than the control diet (*p* < 0.001). A linear reduction of gene expression with the increase in dietary rutin supplementation was detected for *SREBP*-*1c* (*p* < 0.005), *ACCα* (*p* < 0.005), and *SCD1* (*p* < 0.005). A significant quadratic response of *ACCα* expression to the dietary rutin was observed also (*p* < 0.005). The lowest *ACCα* abundance was obtained when the dietary rutin was 0.09% (*p* < 0.05). There was no significant effect of dietary supplementation of rutin on hepatic *FAS* abundance (*p* > 0.05).

## 4 Discussion

While scientific studies have shown that rutin is an effective lipid metabolism modulator for mammals, this excellent metabolic modulatory property has not been fully validated in poultry trials (22, 23). In poultry production, the problem of impaired lipid metabolism in post-peak hens is an essential point in reducing productivity. This is the first study focusing on the effects of rutin on lipid metabolism in post-peak laying hens, and these results provide a theoretical basis and new perspectives for the development of metabolic modulators in laying hens.

From the perspective of egg quality, dietary rutin significantly improves eggshell strength and reduces yolk proportion. It has become accepted that egg quality declines as the hen ages (24). Therefore, the increase in eggshell strength and decrease in yolk specific gravity may be an outward sign of restored production vigor in laying hens (25). As a class of estrogen-liked active substances, rutin may improve eggshell quality by enhancing calcium deposition (26). And the antioxidant function of flavonoids is also one of the reasons why rutin improves egg quality (27), which can be supported by our data. In this study, rutin also reduced the yolk fat and TG content, which was a direct cause of the decrease in yolk specific gravity. Similarly, Liu et al. (28) demonstrated that dietary supplementation of 0.4 g quercetin/kg feed increased eggshell strength and reduced yolk cholesterol content. The level of yolk lipid content is not a judgment of egg quality, but eggs with lower lipid content provide a consumer trend for those who require a low-fat diet (8, 29).

Normally, variations in metabolic and physiological processes tend to affect relevant aspects of animal health (30). When liver cells are damaged by inflammation, toxicity, necrosis, etc., transaminases are released into the bloodstream and serum transaminases are elevated (31). The tendency to increase AST levels due to rutin concentration in the diet, cannot be tied to liver problems. In fact, AST, as a mitochondrial enzyme, is considered a less specific indicator of liver function than ALT since it can also be found in many peripheral tissues such as in the muscles and hence has a very wide variability (32). We also observed significant changes in serum TC, TG and HDL-C levels in hens fed diet with supplementation of rutin. These observations were similar to the findings reported in broilers (33), suggesting that rutin plays an important role in regulating lipid metabolism. A published study has shown that rutin reduces cholesterol synthesis by stimulating *PPARα* and downstream target enzymes, thereby reducing the amount of free fatty acids available for TC synthesis (22), and this viewpoint is in agreement with our experimental data. In addition, beneficial effects of dietary flavonoid supplementation on plasma TG and TC levels were reported in previous studies (28, 34). Therefore, the lipid-lowering function observed in hens fed diet with rutin reflected the nature of its chemical structure as a flavonoid glycoside.

As we hypothesized, supplementation of rutin reduced liver index and PAF in post-peak laying hens. The findings of a previous study indicated that the administration of rutin reversed obesity, dyslipidemia, and hepatic steatosis in rats that had been induced to develop these conditions by a high-fat diet (23). Flavonoids or flavonoid-rich plant extracts reduce hepatic TG levels and fat content by activating the *AMPK* pathway (35, 36). This is consistent with our experimental finding that rutin reduced hepatic lipid (TG, TC and FFA) content in laying hens. In the “2-hit hypothesis” of fatty hepatic disease, liver TG accumulation, increases the liver’s susceptibility to damage mediated by cytotoxic events (mitochondrial dysfunction, oxidative stress, etc.) (37). The biological function of rutin in reducing hepatic lipid accumulation implies that it could attenuate the risk of hepatic steatosis in aged layers, the results that are novel and have not been reported in hens. In addition, as liver is the main organ for lipid metabolism in poultry, the enrichment and activity of lipid metabolizing enzymes directly affect the rate of lipid processing in the liver (38, 39). Indeed, the oxidative capacity and the activity of enzyme systems associated to lipid metabolism measured in this experiment increased in hens fed diets with rutin, demonstrating that the reduced lipid accumulation is associated with the oxidative capacity and the activity of lipid metabolizing enzymes.

Due to prolonged laying activity and aging, reactive oxygen species accumulate in laying hens during the post-peak laying period, and the accumulation can attack polyunsaturated fatty acids in biofilms, causing lipid peroxidation (8). To evaluate the hepatic lipid peroxidation status, we measured hepatic antioxidant enzyme system (T-AOC, SOD, and GSH-Px) activities and lipid peroxide (MDA) levels in this research. Indicator measurements indicated that dietary supplementation of rutin effectively increased the activities of T-AOC and SOD, particularly at the level of 0.09%. In consistent with the increase in activities of T-AOC and SOD, the end product of lipid peroxidation decreased in the liver of the hen fed diets with the supplementation of rutin, suggesting the protective effect of rutin against hepatic lipid peroxidation in aged laying hens. Comparable results were reported also in previous studies with poultry (21, 33). The anti-free radical activity of rutin is mainly attributed to the free hydroxyl groups on C3′, C4′, and C7 (40). And beyond the structural chemistry, rutin also promotes the production of GSH-Px and SOD by activating the *Nrf*2 signaling pathway in order to enhance free radical scavenging and maintain the redox balance of the organism (41). It is noteworthy that the antioxidant indices did not show marked variability between the additive groups, which may be related to the degree of utilization of rutin in monogastric animals, which is a regrettable aspect of this experiment. A reasonable increase in the concentration of the additive and the duration of the test under safe conditions may be an effective way to address this shortcoming.

It is generally accepted that the dysfunction of lipid metabolism is the initial link of hepatocellular steatosis and changes in lipid flux output. Disorders of lipid metabolism, in turn, are usually accompanied by changes in the activity of hepatic lipid metabolizing enzymes. FAS and ACC are essential for the biosynthesis of TG and TC, and are the rate-limiting enzymes for fatty acid production (42, 43). In addition, all the hydrogen required for fatty acid synthesis in the liver is provided by NADPH. In contrast, the NADPH required for fatty acid synthesis in the liver of birds is supplied mainly through the pyruvate-malate pathway, comprising NADP-ME (44). As the major metabolite of rutin, quercetin can increase the potential of anti-steatosis in the liver of rat by reducing the activity of hepatic lipogenic enzymes and activating the cytoplasmic lipase adipose triglyceride lipase (ATGL) activity (45). Administration of rutin in adipocytes inhibits the activity of alpha-glucosidase and pancreatic lipase and increases adiponectin levels *in vitro* (46). To the best of our knowledge, this is the first study on the effect of rutin on hepatic lipid metabolizing enzymes in laying hens, and the results showed that dietary rutin could reduce the activities of ACC and ME in the liver of aged laying hens. The reduction in liposynthase activity could be related to the previously mentioned increase in adiponectin levels. Adiponectin reduces hepatic lipid accumulation by decreasing the expression of genes involved in hepatic lipogenesis and cholesterol synthesis via inhibition of *SREBP*-*1c* expression (47). Interestingly, we also found that dietary rutin simultaneously reduced hepatic HL activity. LPL and HL are key enzymes in lipolysis and are important lipases for TG catabolism (48). The reduction in the activity of LPL and HL is thought to be a side effect of the restoration of lipid metabolic homeostasis, which in turn further normalizes lipolysis (49).

To further explore the regulatory effects of dietary rutin on lipid metabolism in laying hens, we screened seven key genes related to fatty acid oxidation (*CPT-1*, *PPARα*, and *FXR*) and anabolic metabolism (*SREBP-1c*, *ACC*α, *SCD1*, and *FAS*) and evaluated their expression in the liver. Both *CPT-*1 and *PPAR*α play important roles in fatty acid degradation, with *CPT*-1 being the rate-limiting enzyme for fatty acid β-oxidation, and *PPAR*α a transcription factor that promotes fatty acid β-oxidation and lipid oxidation (19, 50). Apart from involving in bile acid metabolism, *FXR* also plays an important role in the metabolism of lipids, carbohydrates and hydrocarbons (51). Increased abundance of hepatic *CPT-1*, *PPAR*α, and *FXR* genes in the present study implied enhanced fatty acid oxidation and hydrolysis in the rutin-added groups, which correlated with restoration of hepatic antioxidant capacity. In recent years, flavonoids have been shown to enhance fatty acid oxidation and fat depletion by upregulating β-oxidation pathway-related genes (*CPT-1*, *PPARα*, and *ATGL*) in mice (36, 52). Similarly, the present study also discovered that the relative expression of *SREBP*-*1c*, *ACC*α, and *SC*D1 were significantly downregulated, which result was consistent with the results of hepatic metabolic enzyme activities. What is certain is that *SREBP-1c* is able to regulate lipogenesis by changing the expression level of its own mRNA, i.e., the transcriptional regulation of hepatic lipid synthase genes is controlled by the amount of *SREBP-lc* mRNA (44). Parallel to this, *SCD*1 is the rate-limiting enzyme in the synthesis of monounsaturated fatty acids, which directly affects the synthesis of TG and TC (53). Hassan et al. (19) also posted a report that rutin reduces lipogenesis and accumulation by downregulating the expression of *ACC* and *FAS*. In short, rutin reduces lipogenesis and promotes lipolysis in old laying hens by decreasing the rate of fatty acid synthesis and accelerating fatty acid β-oxidation, which responds to positive changes in egg quality.

## 5 Conclusion

Our results suggest that the addition of 0.06% ~ 0.12% rutin to the diets of post-peak laying hens under the conditions of this experiment effectively reduces serum lipid levels, inhibits lipid peroxidation, regulates lipid metabolism, and reduces the deposition of liver and abdominal fat, 0.09% of rutin played a more effective role. This finding to some extent supports the application of rutin as a lipid metabolism regulator and production promoter in laying hens, and provides a theoretical reference for the promotion of healthy and sustainable development of poultry.

## Data availability statement

The original contributions presented in the study are included in the article/supplementary material, further inquiries can be directed to the corresponding authors.

## Ethics statement

The animal study was approved by Institutional Animal Care and Use Committee of Hebei Agricultural University. The study was conducted in accordance with the local legislation and institutional requirements.

## Author contributions

LZ: Data curation, Writing – original draft, Writing – review & editing. JG: Validation, Writing – review & editing. LX: Writing – review & editing. BY: Writing – review & editing. YH: Writing – review & editing. HZ: Writing – original draft. ZF: Data curation, Writing – review & editing. QL: Writing – review & editing.
